# 578 Impact of Acellular Fish Skin on Wound Healing in a Standardized Pre-Clinical Model

**DOI:** 10.1093/jbcr/irad045.173

**Published:** 2023-05-15

**Authors:** Anna-Lisa Pignet, Elisabeth Hofmann, Julia Fink, Anna Schwarz, David Hahn, Manuel Prevedel, Lars-Peter Kamolz, Petra Kotzbeck

**Affiliations:** JOANNEUM RESEARCH Forschungsgesellschaft mbH & Medical University of Graz, Graz, Steiermark; JOANNEUM RESEARCH Forschungsgesellschaft, Graz, Steiermark; Joanneum Research, Graz, Steiermark; Joanneum Research, Graz, Steiermark; Joanneum Research COREMED, Graz, Steiermark; COREMED – Kooperatives Zentrum für Regenerative Medizin, Graz, Steiermark; Medical University Graz and Joanneum Research Forschungsgesellschaft mbH, Graz, Steiermark; JOANNEUM RESEARCH Forschungsgesellschaft & Medical University of Graz, Graz, Steiermark

## Abstract

**Introduction:**

Acellular fish skin has proven to be effective in acute and chronic wound healing. Multiple human studies have to date demonstrated that products from acellular skin grafts accelerate wound healing and are efficacious, safe and cost-effective. Here we wanted to investigate the effects of acellular fish skin on wound healing in a pig model.

**Methods:**

The primary aim of this study was to investigate the effects of acellular fish skin on wound healing progression in a full thickness skin defect pig model. The superiority of fish skin was tested in a standardized pig model and compared to normal wound healing (without acellular fish skin grafts) (n=6).

The experiments were carried out in male landrace pigs with a mean weight of 28.8 +/- 2.8 kg at the start of the study. The experiment lasted for 21 days. Wound documentation, thermography, photo-documentation and wound perfusion were carried out 5, 9, 14 and 21 days post wounding.

**Results:**

Clinical evaluation of the wounds showed that there were no major differences between the different dressings on day 5 post wounding. On the next evaluation days ( day 9, 14 and 21 post wounding) the acellular fish skin showed rapid epithelialization with minimal wound contraction, when compared to untreated control wounds, which showed significant signs of contraction (i.e. star-like wound appearance).

Wound area measurements clearly showed that wound size upon treatment with the acellular fish skin and the untreated control rapidly and significantly decreased through contraction of untreated controls. However, wound perfusion seemed to be affected by the treatment with acellular fish skin. Thermography at day 9 and 14 showed higher surface temperature in treated wounds (d9 and d14; p = 0.0005), followed by a trend towards increased oxygenation and perfusion, analyzed by laser speckle and hyperspectral imaging.

**Conclusions:**

Acellular fish skin seems to provide fast wound healing and counteract wound contraction. Untreated wounds showed a slight trend of faster healing, but showed significant signs of wound contraction which points towards wound healing by contraction but not by epithelialization. This contraction was not observed in wounds treated with acellular fish skin.

**Applicability of Research to Practice:**

Standardized preclinical models give valuable insights to the mechanisms of wound healing. Porcine in vivo models are of special interest as the porcine skin is highly similar to human skin with regard to anatomical and physiological structures. Therefore, data generated in such models, as described here, show a high rate of translatability to the human situation.

